# Usefulness of Thulium-Doped Fiber Laser and Diode Laser in Zero Ischemia Kidney Surgery—Comparative Study in Pig Model

**DOI:** 10.3390/ma14082000

**Published:** 2021-04-16

**Authors:** Bogusława Żywicka, Jolanta Bujok, Maciej Janeczek, Albert Czerski, Maria Szymonowicz, Maciej Dobrzyński, Jacek Świderski, Zbigniew Rybak

**Affiliations:** 1 Department of Experimental Surgery and Biomaterial Research, Wroclaw Medical University, Bujwida 44, 50-368 Wroclaw, Poland; boguslawa.zywicka@umed.wroc.pl (B.Ż.); maria.szymonowicz@umed.wroc.pl (M.S.); zbigniew.rybak@umed.wroc.pl (Z.R.); 2 Division of Animal Physiology, Department of Animal Physiology and Biostructure, Wroclaw University of Environmental and Life Sciences, C.K. Norwida 31, 50-375 Wroclaw, Poland; albert.czerski@upwr.edu.pl; 3 Division of Anatomy, Department of Animal Physiology and Biostructure, Wroclaw University of Environmental and Life Sciences, Kożuchowska 1, 51-631 Wroclaw, Poland; maciej.janeczek@upwr.edu.pl; 4 Department of Pediatric Dentistry and Preclinical Dentistry, Wroclaw Medical University, Krakowska 26, 50-425 Wroclaw, Poland; maciej.dobrzynski@umed.wroc.pl; 5 Institute of Optoelectronics, Military University of Technology, Kaliskiego 2, 00-908 Warsaw, Poland; jacek.swiderski@wat.edu.pl

**Keywords:** thulium-doped fiber laser (1940 nm), diode laser (1470 nm), partial nephrectomy, zero ischemia, porcine model, hemostasis, thermal damage zone

## Abstract

Background: The aim of this study was to evaluate the usefulness of a thulium-doped fiber laser and a diode laser in zero ischemia kidney surgery, by carrying out a comparative study in a pig model. Material and methods: Research was carried out on 12 pigs weighing 30 kg each. A thulium-doped fiber laser (TDFL) and a diode laser (DL) operating at wavelengths of 1940 and 1470 nm, respectively, were used. The cut sites were assessed both macroscopically and microscopically. The zone of thermal damage visible in the histopathological preparations was divided into superficial and total areas. Results: During partial nephrectomy, moderate to minimal bleeding was observed, which did not require additional hemostatic measures. All animals survived the procedure. On day 0, the total thermal damage depth was 837.8 µm for the TDFL and 1175.0 µm for the DL. On day 7, the depths were 1556.2 and 2301.7 µm, respectively. On day 14, the overall thermal damage depth for the DL was the greatest (6800 µm). The width of the superficial zone was significantly reduced on days 7 and 14 after TDFL application. Conclusion: Both lasers are suitable for partial wedge nephrectomy without ischemia in pigs. The TDFL produced similar or better hemostasis than the DL, with a smaller zone of thermal damage and, therefore, seems more suitable for application in human medicine.

## 1. Introduction

The widespread use of ultrasound examination and computed tomography (CT) scanning for the evaluation of abdominal symptoms not related to kidney disease has been associated with a significant increase in the percentage of renal tumors incidentally found at an asymptomatic stage. These tumors are usually small lesions, suitable for elective partial nephrectomy [1,2]. Partial nephron-sparing nephrectomy in these patients is a therapy of choice. An optimal surgical technique for partial nephrectomy should provide precise tumor excision with minimal or no bleeding. The conventional laparoscopic procedure significantly decreases the amount of intraoperative blood loss. However, the main disadvantage of this method is the longer duration of the operation, associated with longer warm renal ischemia due to hilar clamping [3]. Most advanced oncology centers have specialized in robotic partial nephrectomy. This technique ensures great precision and short recovery time. However, hilar clamping remains a part of the procedure and sophisticated methods of very selective arterial clamping may be difficult to reproduce [4]. Ischemia of the remaining renal tissue is of concern, mainly in patients with concomitantly reduced kidney function. Cutting off the arterial blood supply for just a few minutes has been associated with a significant production of free radicals after reperfusion of hypoxic kidney cells, which are typically characterized by intensive oxygen metabolism [5]. This leads to damage to the kidney parenchyma and, potentially, to a reduction in glomerular filtration. In patients with chronic kidney disease, this may result in worsening azotemia, thus shortening survival due to progression to end-stage kidney disease [6]. Therefore, further efforts are being made to develop modifications to existing surgical procedures or new techniques that shorten the time of warm renal ischemia or entirely eliminate the need for hilar clamping.

Lasers have widely been used in surgery, as well as in urological and endourological procedures. This is due to the potential for simultaneous tissue cutting, coagulation, and hemostasis. Principally, the effects of a surgical laser depend on the amount of the corresponding chromophore [7]. Absorption of laser light results in the production of heat and, thus, tissue coagulation, hemostasis, evaporation with a cutting effect, and carbonization, depending on the temperature. In a perfect case, the laser used for a partial nephrectomy should produce a strong point thermal effect, allowing for precise cutting with a clean margin and a limited local thermal effect, leading to effective hemostasis with minimal thermal damage to the adjacent tissue [8].

The most commonly used lasers applied in medical applications are gas lasers operating in the far infrared spectral region, as well as solid-state lasers providing emission at wavelengths in the range of 1–3 µm. A molecular CO_2_ laser, being the main representative of gas lasers, operates at 10.6 µm (basic emission linewidth) and provides continuous wave (CW) high power (even kW-level) output radiation, being suitable for surgery [9]. However, due to the fast degradation of the gas active media, their operating time is short, which significantly increases the associated maintenance costs. Furthermore, the radiation emitted by such lasers cannot be guided to the place of interaction by conventional treatment fiber probes, which is a significant disadvantage. The next important tool for medical applications is a flash-lamp pumped Er:YAG laser, emitting at a wavelength of ~2.94 µm [10]. Laser radiation of this wavelength corresponds to a strong absorption peak in soft and hard biological tissues (~10,000 cm^−1^) containing a significant amount of water, which makes erbium lasers an effective surgical tool. They can operate in pulse mode, delivering mJ optical pulses at a repetition rate of up to several dozens of Hz. The light at ~2.94 µm can be transmitted by sapphire fibers; however, these are expensive and cannot be sterilized in autoclaves. Moreover, due to the high absorption of light at ~2.94 µm by laser components (e.g., mirrors, gain medium, and electro-optic cells) the device is susceptible to damage, which entails high maintenance costs. Lasers operating at a wavelength of ~2 µm—namely, Ho:YAG (λ = 2.12 µm) [11] and Tm:YAG (λ = 2.01 µm) [12], among others—are also good candidates for medical applications. The radiation emitted by these lasers can be transmitted by a variety of commercially available silica-based fibers with reduced OH ions. The possibility of working in CW, pulsed, and quasi-CW modes of operation is another advantage. They can be both flash- and diode-pumped. In particular, diode-pumping offers very good performance with lower thermal load, making the devices more reliable. Diode-pumped thulium-doped fiber lasers operating at a wavelength of 1.94 µm have been also successfully used for soft tissue surgery [13,14]. Their unique feature is the possibility of developing in an all-fiber architecture, thus making the whole laser system resistant to dust, vibration, and moisture. Furthermore, these devices can provide a single-mode beam of high quality, which can be emitted from a fiber core with diameter of 25 µm or less. This means that such a beam can be easily launched into the thinner fiber probes used in robotic endoscopes and surgical robots. Fiber-pigtailed high-power laser diodes (operating at wavelengths of 1470 and 980 nm) are also worth mentioning [15]. Although these semiconductor lasers emit multimode beams of low spatial quality, they can be run in a quasi-CW mode, delivering high energy pulses which are useful for operations in organs with high blood flow, such as the liver, spleen, and venous system. All the lasers mentioned above have advantages and disadvantages, and their choice is strictly determined by a particular application.

Over the past two decades, more and more attention has been paid to the use of lasers in partial nephrectomy. Experiments have been carried out on animal models using potassium-titanyl-phophate, holmium:YAG, and thulium:YAG lasers, often with promising results [16,17,18,19]. However, in human medicine, laser nephrectomy is still in clinical trials. In the past few years, small retrospective studies on human patients have been published, presenting the results of both open and laparoscopic partial nephrectomy with diode and thulium lasers [2,20,21,22,23,24]. Recently, thulium laser-assisted laparoscopic partial nephrectomy without hilar clamping for small renal mass has been shown to be superior to conventional laparoscopic surgery, in terms of early postoperative changes in glomerular filtration [24]. Moreover, a thulium laser has been evaluated and seemed to be a promising tool in robotic partial nephrectomies [25].

As lasers are becoming more and more important in nephron-sparing renal surgery, the aim of our study was to compare a newly developed thulium-doped fiber laser and a diode laser, in terms of their cutting efficacy, hemostasis, and early and delayed tissue thermal damage in a pig model of open zero-ischemia kidney incision and partial nephrectomy.

## 2. Materials and Methods

### 2.1. Lasers

In the experiment, two surgical lasers were compared. A thulium-doped fiber laser (TDFL) and a diode laser (DL), operating at wavelengths of 1940 and 1470 nm, respectively, were used. Both lasers were constructed and manufactured by Metrum Cryoflex Sp. z o. o. (Blizne Łaszczyńskiego, Poland) and the Military University of Technology.

The thulium-doped laser used in the experiment was a single-mode device with a maximum output power of 34.7 W at a wavelength of 1970 nm. The laser was constructed with a double-clad fiber, doped with thulium ions (Tm^3+^), and with a core/clad diameter of 25/250 μm. The beam quality for the output radiation was of high quality, allowing for its use in thin treatment probes (less than 50 µm in diameter). The design and detailed physical parameters of the laser have been described in detail elsewhere [8,26,27].

The diode laser was designed as a multimodal semiconductor laser working in a continuous (CW) and quasi-continuous (QCW) mode. Its maximum power was 100 W at a wavelength of 1470 nm. The laser beam was placed directly in the laser probe with a diameter of 400 μm.

In the course of the preliminary experiments, the optimal cutting parameters for each laser were determined. Laser probes were held in direct contact with the tissue surface during cutting. Both thulium and diode lasers were operated in CW mode with constant parameters, as listed in Table 1.

### 2.2. Experimental Animals

The experiments were performed on 12 female pigs aged 10 weeks. The animals were purchased from a certified farm (National Research Institute of Animal Production in Pawłowice) and were subjected to a 2-week acclimatization before the experiments. The pigs were clinically healthy and weighed approximately 30 kg. All procedures were approved by the II Local Ethical Committee in Wrocław (Approval No. 87/2012) and performed in accordance with the EU Directive (2010/63/EU). Animals were allocated into two equal experimental groups, T7 and T14, depending on the time after surgery at which they were euthanized. In each animal, cuts with both lasers were performed and partial nephrectomy was done with one type of laser.

### 2.3. Surgery

The animals were fasted and clinically healthy on the day of surgery. They were sedated by intramuscular administration of a mixture of medetomidine and butorphanol (0.1 mg/kg body weight, Domitor^®^, Orion Pharma, Warsaw, Poland; 0.2 mg/kg body weight, Butomidor^®^, Richter Pharma AG, Wels, Austria). They were placed on the operating table in supine position. Intravenous access was obtained by inserting an intravenous catheter into the marginal vein of the ear and general anesthesia was induced by bolus administration of propofol (4 mg/kg, Scanofol^®^, Scan Vet Sp. z o. o., Skiereszewo, Poland). The animals were intubated and anesthesia was maintained with an isoflurane/oxygen mixture. Analgesia was provided by continuous intravenous infusion of fentanyl (500 μg/h, Polfa SA, Łódź, Poland). After the procedure and for the next 3 days, the animals were administered amoxicillin (15 mg/kg body weight, Betamox LA^®^, ScanVet Sp. z o. o., Skiereszewo, Poland) and metamizol (30 mg/kg body weight, Biovetalgin^®^, Biowet Drwalew, Drwalew, Poland).

Surgical access to the left kidney was achieved by a midline incision of the abdominal wall. After cutting the abdominal integuments (skin, subcutaneous tissue, rectus abdominis sheath, and peritoneum), the intestines were displaced head and laterally from the kidney, securing them with gauze soaked in natrium chloratum 0.9%. The kidney was lifted and the blood vessels (renal artery and vein) were located for possible temporary closure, if intraoperative bleeding occurred during laser use. In each animal, transverse cuts were performed in the middle part of the lateral edge of kidney, both with the TDFL and DL. Moreover, on the caudal pole of the kidney, a small wedge-shaped fragment was cut out using the TDFL or DL. The width of the wedge was about 1.5 cm which was approximately 1 cm deep, corresponding to kidney tumor size classified as T1, according to Preoperative Aspects and Dimensions Used for an Anatomical (PADUA) classification of renal tumors [1]. During partial nephrectomy, no clamping of the hilar vessels was used (Figures S1 and S2). Excised fragments were immediately harvested for microscopic analysis. During the procedure, cutting efficacy and bleeding were assessed. After the procedure, the abdominal wall was closed with three layers of sutures (muscles and peritoneum 3–0 absorbable sutures, subcutaneous 3–0 absorbable sutures, skin 2–0 non-absorbable sutures) and animals were weaned from anesthesia.

### 2.4. Macroscopic Evaluation

Intraoperatively, the macroscopic appearance of the incision and the wedge nephrectomy sites was assessed. Moreover, bleeding from the incision and resection site was semi-quantified: three pluses (+ + +) were assigned for visible moderate bleeding, (+ + −) for light bleeding, (+ − −) for minimal bleeding, and (− − −) for no bleeding. On days 7 and 14, the gross appearance of the wound healing and the shape of the scar after wedge excision was examined.

### 2.5. Microscopic Evaluation

Kidney fragments were prepared as described previously [26,27]. Briefly, excised fragments and portions of the organ at the site of excision were fixed in 10% buffered formaldehyde solution and then cut into small blocks transversely to the cutting line and embedded in paraffin. Approximately 4 μm-thick sections (Leica 2025 rotational microtome, Leica Microsystems, Wetzlar, Germany) were prepared and stained with hematoxylin and eosin (H&E; Sigma-Aldrich, Saint Louis, MO, USA). The microscopic appearance of the kidney was evaluated using a light microscope (Olympus BX43, Olympus Corporation, Tokyo, Japan) and acquisition imaging software (Version 1.6; 2010 Olympus Corporation, Tokyo, Japan).

### 2.6. Statistics

Data are expressed as mean ± standard deviation for n animals. For the analysis, an average from measurements carried out in five to seven stained slides for each animal was used. Data were analyzed by Student’s *t*-test using the Statistica Version 10.0 software package (StatSoft, Tulsa, OK, USA). Differences between means were considered significant when *p* ˂ 0.05.

## 3. Results

### 3.1. Intraoperative Bleeding Assessment and Surgical Outcome

**Figure 1:**
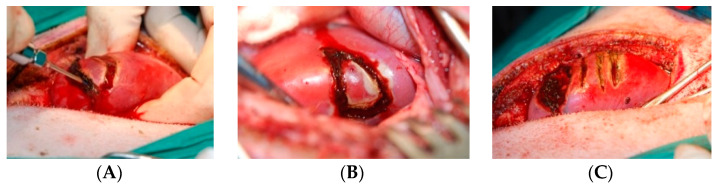
Intraoperative assessment of bleeding and thermal damage during wedge nephrotomy and kidney incision with a TDFL and DL: (**A**) wedge nephrectomy with DL; (**B**) wedge nephrectomy with TDFL; (**C**) two transverse incisions, the TDFL incision on the left and DL on the right; the left wound is pictured after wedge nephrectomy with TDFL.

Minimal (+ − −) or no bleeding (− − −) was observed during cutting of the kidney in the middle part of the lateral margin. Wedge excision with both the TDFL and DL was associated with transient (+ + +) to (+ − −) bleeding from the cutting site. However, partial nephrectomy with the DL typically produced more intense bleeding (usually + + −) than with the TDFL. No prolonged bleeding from the wounds after cutting was observed during surgery and no additional hemostatic agents were required (Figure 1). Extravasated blood volume was less than 50 mL in all pigs. All animals were successfully weaned from anesthesia and survived until euthanasia in a good clinical condition.

### 3.2. Macroscopic Evaluation

**Figure 2:**
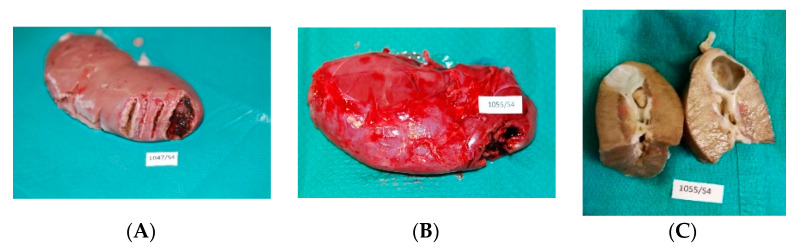
Macroscopic appearance of the kidneys on day 7 (**A**) and 14 (**B**,**C**) after surgery: (**A**) partially healed wounds after (from the left side) incision with DL, TDFL, and partial nephrectomy with TDFL. (**B**) A small recess shown in a kidney after partial nephrectomy on day 14; and (**C**) a cystic wedge-shaped lesion visible after cutting the organ in a long axis on day 14.

Intraoperatively, a visible zone of carbonization was produced by both lasers; however, the TDFL seemed to produce a narrower zone of thermal damage (Figure 1). Seven days after surgery, the kidneys had normal gross appearance. Partially healed wounds after incisions were visible on the surface of the organ, along with light streaks of burn lesions, which were wider after cutting with the DL than with the TDFL (Figure 2). The kidneys were macroscopically normal 14 days after surgery. Sites of incisions with the TDFL and the DL were poorly visible. At the site of the wedge nephrectomy, a small recess or discoloration on the continuous surface was visible. There were no macroscopic lesions in the immediate vicinity of the excision sites. In an individual case, after cutting the kidney in a long axis, a cyst filled with serous fluid was visible (Figure 2).

### 3.3. Microscopic Evaluation

Histologically, the wound healing process was comparable after excision of the wedge-shaped fragment with the TDFL and DL; however, the thermal damage zone was narrower for the TDFL.

**Figure 3:**
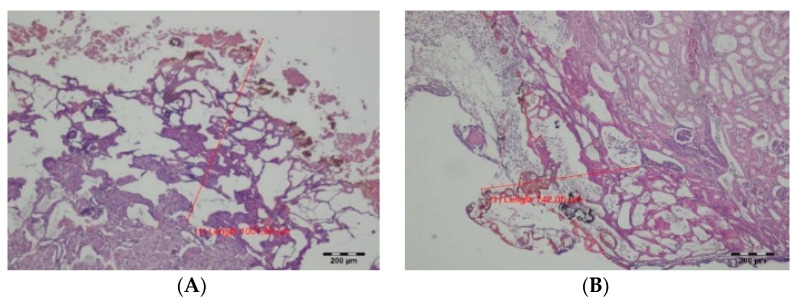
Histological features of the excised fragments—intraoperative thermal damage caused by: (**A**) DL, magnification: 40× and (**B**) TDFL, magnification: 40×.

In excised tissue fragments, immediate thermal changes were seen as a carbonization zone with a poorly expressed exudative phase with a small number of erythrocytes. Deeper, a zone of shrinkage was present, with cells that had preserved nuclei. This zone was sharply demarcated from normal tissue. The TDFL produced a slightly narrower zone of carbonization, compared to the DL. Similarly, the total depth of the thermal damage zone (TDZ) was approximately 873.20 µm after using the TDFL, while the DL produced a TDZ of 1174.96 µm (Table 2; Figure 3).

**Figure 4:**
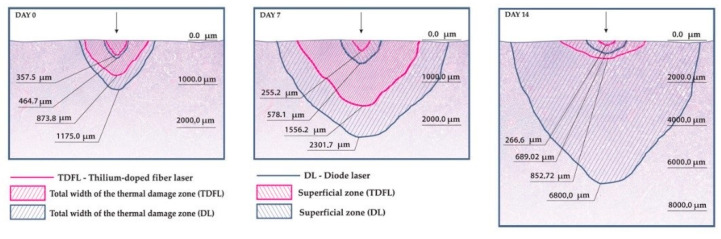
Scheme of the depth of all thermal damage in the renal cortex of pigs after incision with a thulium-doped fiber laser and diode laser.

**Figure 5:**
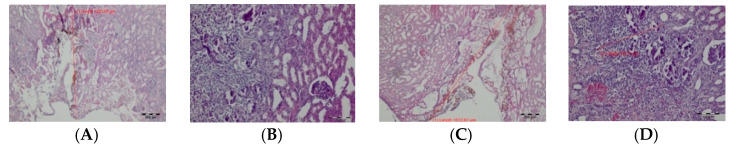
Zones of thermal damage in the renal cortex after cutting with TDFL and DL on day 7: (**A**) thermal damage zone produced by TDFL, magnification: 40×; (**B**) border between thermal damage zone and healthy tissue in a sample from the kidney cut with TDFL, magnification: 400×; (**C**) thermal damage zone produced by DL in the renal cortex, magnification: 40×; and (**D**) deeper thermal changes after cutting with DL, magnification: 100×.

On day 7, the remains of the exudative phase and fragments of brown carbonized tissues were visible along the cutting line. In case of incisions, thermal changes were arranged in a semicircle around the cutting axis. The depth of the lesions was measured in places where the laser was acting at right angles to the tissue surface (Figure 4). The wound was filled with granulation tissue containing regenerating renal cortical tissue and young highly cellular connective tissue. Deeper, a semicircular band of connective tissue with numerous proliferating fibroblasts and focal necrotic lesions was visible. These changes were sharply separated from the normal parenchyma of the renal cortex (Figure 5). The average depth of the superficial changes was 255.21 µm and total depth of TDZ was 1556.90 µm in tissues cut with the TDFL. The depth of superficial and total TDZ after cutting with the DL were 578.12 and 2301.39 µm, respectively (Table 2).

**Figure 6:**
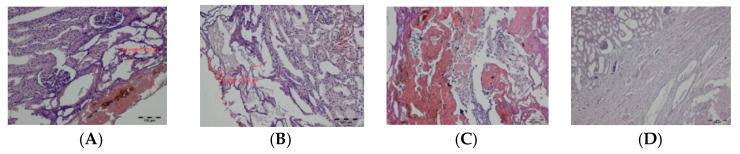
Zones of thermal damage in the renal cortex on day 14 after cutting the kidney with TDFL and DL: (**A**,**B**) superficial zone of thermal damage in renal cortex after cutting with TDFL, magnification: 40×; (**C**) thermal damage in renal cortex after cutting with DL, magnification: 100×; and (**D**) deeper thermal changes in renal cortex after cutting with DL, magnification: 100×.

In tissues obtained 14 days after surgery, with both the TDFL and DL the superficial zone of thermal damage was characterized by the presence of thin strands of fibrous connective tissue. Where the laser beam was perpendicular to the surface of the organ, the remains of the exudative phase (in the form of brown homogeneous masses) were present, along with the remains of carbonized tissues. From the edges, the wound was filled with a semicircular fibrous connective tissue with the remains of carbonized necrotic structures and associated inflammatory cells, forming the deeper zone of thermal damage. This band was sharply demarcated from the normal kidney parenchyma. On the border with the normal kidney cortex, focal disappearing necrotic changes were visible (Figure 6). Both the superficial and total zones of thermal damage were deeper after using the DL (Table 2).

## 4. Discussion

Research on the use of lasers in partial nephrectomy has focused on obtaining both precise cuts and optimal hemostasis, in order to avoid the clamping of renal vessels and, thus, organ ischemia.

In our study, incision of the left kidney margin did not result in significant bleeding, regardless of the laser used. Moreover, partial wedge nephrectomy at the caudal pole of the organ without hilar clamping typically produced only minimal transient bleeding. In particular, the TDFL produced good hemostasis.

Our results suggest that a TDFL operating at 1940 nm allows for better bleeding control. Similar results were obtained in experiments on the application of a thulium laser in laparoscopic partial nephrectomy without hilar clamping in a pig model [20]. The estimated blood loss in animals did not exceed 50 mL during the procedure and the use of additional hemostatic agents was not required. It was found that the thulium laser working in continuous mode with a power of 30 W was able to coagulate vessels up to 1.6 mm in diameter [20]. Similarly good hemostasis has been reported in another pig model study on the feasibility of a thulium:YAG laser, which operates at slightly higher wavelength compared to the TDFL, for laparoscopic partial nephrectomy [16]. Moreover, a thulium laser operating at 1920 nm was found to be comparable to a high-frequency dissection device, in terms of hemostasis during wedge nephrectomy and heminephrectomy in a porcine model [28]. A retrospective study in human patients showed that a 2000 nm continuous thulium laser at 30–40 W may be used for small renal mass excision without renal hilum clamping [24]. However, in some cases, additional hemostatic agents were necessary [24]. Another report of four cases proved the feasibility of a 2013 nm thulium laser with output power of 30 W for the excision of renal cortical tumors during open surgery without renal artery clamping. The mean blood loss related to the procedure was 65 mL [23]. Moreover, a thulium:YAG laser allowed for the excision of small exophytic renal tumors with zero ischemia during the procedure [29]. We studied both lasers in an open surgery model; however, thulium lasers are also promising for laparoscopic procedures. Zero ischemia with negative tumor margins has been reported in human patients [29]. In a porcine model of laparotomic partial nephrectomy, a thulium laser produced minimal smoke and provided excellent precision [20]. There have also been preliminary reports on the feasibility of use of a thulium laser for zero ischemia robot-assisted excision of small kidney tumors [25]. Therefore, it seems that this type of laser can be increasingly applied in kidney surgery, along with the use of the most advanced surgical approach techniques.

To our knowledge, there have only been two studies on the applicability of diode lasers for partial nephrectomy in a pig model [18,30]. A diode laser operating at 980 nm (23 W) has been used to perform laparoscopic partial excision of the right kidney. In three of five cases, additional hemostatic clips were necessary to stop bleeding and the mean blood loss was 150 mL. The diode laser used in the latter study operated at a shorter wavelength than that tested by us. The 980 nm wavelength corresponds to the absorbance range for hemoglobin [18]. This was probably the reason why the extravasated blood blocked further hemostatic effects of the laser. On the other hand, in an experiment with a diode laser emitting light with a wavelength of 1318 nm, adequate hemostasis was obtained without the need to clamp kidney vessels in pigs, where the mean blood loss was only 30 mL [30]. In studies on the use of diode lasers in human nephrectomy, it has also been observed that lasers operating at longer wavelengths give better hemostatic effects, whether in combination with a 980 nm laser as a dual laser at 980/1470 nm or as a device operating at one wavelength of 1318 nm [2,22].

In contrast to our results, several lasers—holmium:YAG, potassium-titanyl-phosphate, and CO_2_ lasers—have been characterized by less optimal hemostasis. In these cases, either hilar clamping or additional hemostatic agents were used [17,19].

The thulium-doped fiber laser and diode laser used in our study operated at wavelengths of 1940 and 1470 nm, respectively. Both of these wavelengths are in the range absorbed by water. The TDFL laser, however, operates at a wavelength corresponding to the absorbance peak for water [31]. The kidney, like other parenchymal organs, is well-hydrated; for example, in pigs, the maximum absorbance of the liver has been shown to be at 1940 nm and a thulium-doped fiber laser provided a precise cut with good hemostasis in liver parenchyma resection in pigs and humans [13,27,32]. Comparing results, it can be seen that the best hemostatic effect during partial nephrectomy was obtained when the laser wavelength corresponded to the range absorbed by water, between 1300 and 2100 nm. On this basis, it can be assumed that the maximum kidney absorption may fall within this range. Relatively high absorption of the laser beam results in a more precise cut and prevents too deep or uncontrolled penetration of the renal tissue. Combined with continuous wave mode of operation, such lasers produce a moderately wide range of thermal damage to the tissue, seen as coagulation and closure of small vessels. Lasers operating at wavelengths corresponding to hemoglobin absorption are not the optimal choice for kidney surgery, as the extravasating blood impairs their function. On the other hand, lasers with very good cutting properties typically produce a too narrow zone of thermal damage and, thus, insufficient hemostasis.

Both lasers used in our experiment produced moderately narrow superficial zones of thermal damage immediately after surgery, on day 7, and on day 14. However, when comparing the total zones of thermal damage, the DL produced deeper tissue damage. Considering that the bleeding with both lasers was comparable and that the TDFL tended to ensure better hemostasis, it can be assumed that the deeper tissue damage by the diode laser is unfavorable. In our previous reports, we have shown that the thulium-doped fiber laser can efficiently cut parenchymal organs in pigs (i.e., liver and spleen), while producing minimal zones of thermal damage sufficient for hemostasis (maximum depth: 1157.5 μm on day 7 for spleen and 1818.7 μm on day 7 for liver) [26,27].

In our current study, the thermal damage due to the TDFL was the most pronounced on day 7 as well (1556.2 μm). Other researchers have reported comparable values for porcine renal tissue after using the thulium-fiber laser. The thermal damage zone was less than 2 mm 4–6 weeks after the procedure [28]. The diode laser used in our report produced a deeper zone of thermal damage, ranging from 1175.0 μm on day 0 to 6800 μm on day 14. In another study on the use of a diode laser for partial nephrectomy in a porcine model, the entire depth of the thermal damage zone was 3690 μm after four weeks [30]. The extent of the damage may be important for the precision of the surgical incision and maintaining an appropriate margin when excising renal mass. The deep penetration of thermal changes may cause damage to deeper-lying large vessels or the kidney’s collecting system.

However, it should be mentioned that the classical approach to quantification of laser-induced changes may underestimate the real depth of thermal damage. In ex vivo studies on skin and bone cartilage treated with different types of lasers, confocal microscopy imaging revealed deeper changes, as compared to histopathology [33,34]. This technique allows for the visualization of cell viability. It has also been discussed that tissue preparation for histopathological staining may induce shrinkage of the thermally injured area and, thus, decrease the width of the damaged tissue [34].

## 5. Conclusions

Both the thulium-doped fiber laser and diode laser considered were suitable for cutting small fragments of the kidney, in the form of a wedge corresponding to T1a and 1b tumors, according to the PADUA classification without ischemia. The TDFL produced similar or better hemostasis with a smaller zone of thermal damage than the diode laser and, therefore, seems more promising for application in human medicine.

## Figures and Tables

**Table 1:** Parameters of the thulium-doped fiber laser and diode laser used for the incision and partial excision of renal tissue of pigs.

Laser Parameter	Thulium-Doped Fiber Laser	Diode Laser
Wavelength	1940 nm	1470 nm
Mode	CW	CW
Output power	23.5 W	50 W
Laser probe characteristics (Φ and NA)	400 μm; 0.22	400 μm; 0.22
Tissue exposure	Laser probes held in direct contact with cut tissue; incision made with a constant speed of 2–3 mm/s	

**Table 2:** Depth of thermal damage in the renal cortex of pigs after cutting with a thulium-doped fiber laser and diode laser.

Thermal Changes	Thulium-Doped Fiber Laser			Diode Laser		
	T0 (n)	T7 (n)	T14 (n)	T0 (n)	T7 (n)	T14 (n)
**Superficial Zone of Thermal Damage (μm)**	357.5 ± 71.1 (3)	255.2 ± 41.9 (6)^a^	266.6 ± 266.6 (6)^a^	464.7 ± 106.3 (5)	578.1 ± 89.0 (6)^b^	689.02 ± 57.8 (6)^b^
**Entire Zone of Thermal Damage (μm)**	873.8 ± 78.7 (3)^a^	1556.2 ± 113.9 (6)^a^	852.7 ± 93.5 (6)^a^	1175.0 ± 92.9 (5)^b^	2301.7 ± 156.9 (6)^b^	6800 ± 860.2 (6)^b^

Data are expressed as mean ± standard deviation (SD); n, number of animals from which tissues were measured; ^a,b^, different superscript letters in a row denote significant differences between the depth of thermal damage from two lasers at the same time after surgery.

## Data Availability

The data presented in this study are available on request from the corresponding author.

## Supplementary Materials

The following are available online at https://www.mdpi.com/article/10.3390/ma14082000/s1, Figure S1: Kidney incision with a thulium-doped fiber laser, Figure S2: Porcine kidney after laser partial nephrectomy.
