# Supplementary material for: Usefulness of Thulium-Doped Fiber Laser and Diode Laser in Zero Ischemia Kidney Surgery—Comparative Study in Pig Model
Source: Materials (Basel). 2021 Apr 16;14(8):2000. doi: 10.3390/ma14082000 (PMC8073520; doi:10.3390/ma14082000)
Supplement: Supplementary file 1 [file materials-14-02000-s001.zip › materials-1115944-supplementary.pdf]

materials

MDPI

Supplementary

Usefulness of thulium-doped fiber laser and diode laser in zero ischemia kidney surgery—comparative study in pig model

Bogusława Żywicka[1], Jolanta Bujok [2,*], Maciej Janeczek [3], Albert Czerski [2], Maria Szymonowicz [1], Maciej Dobrzyński [4], Jacek Świderski [5] and Zbigniew Rybak [1]

[1] Department of Experimental Surgery and Biomaterial Research, Wroclaw Medical University, Bujwida 44, 50-368 Wroclaw, Poland; boguslawa.zywicka@umed.wroc.pl (B.Z.); maria.szymonowicz@umed.wroc.pl (M.S.); zbigniew.rybak@umed.wroc.pl (Z.R.)
[2] Department of Animal Physiology and Biostructure, Division of Animal Physiology, Wroclaw University of Environmental and Life Sciences, C.K. Norwida 31, 50-375 Wroclaw, Poland; albert.czerski@upwr.edu.pl
[3] Department of Animal Physiology and Biostructure, Division of Anatomy, Wroclaw University of Environmental and Life Sciences, Kożuchowska 1, 51-631 Wroclaw, Poland; maciej.janeczek@upwr.edu.pl
[4] Department of Conservative Dentistry and Pedodontics, Wroclaw Medical University, Krakowska 26, 50-425 Wroclaw, Poland; maciej.dobrzynski@umed.wroc.pl
[5] Institute of Optoelectronics, Military University of Technology, Kaliskiego 2, 00-908 Warsaw, Poland; jacek.swiderski@wat.edu.pl
* Correspondence: Jolanta.bujok@upwr.edu.pl

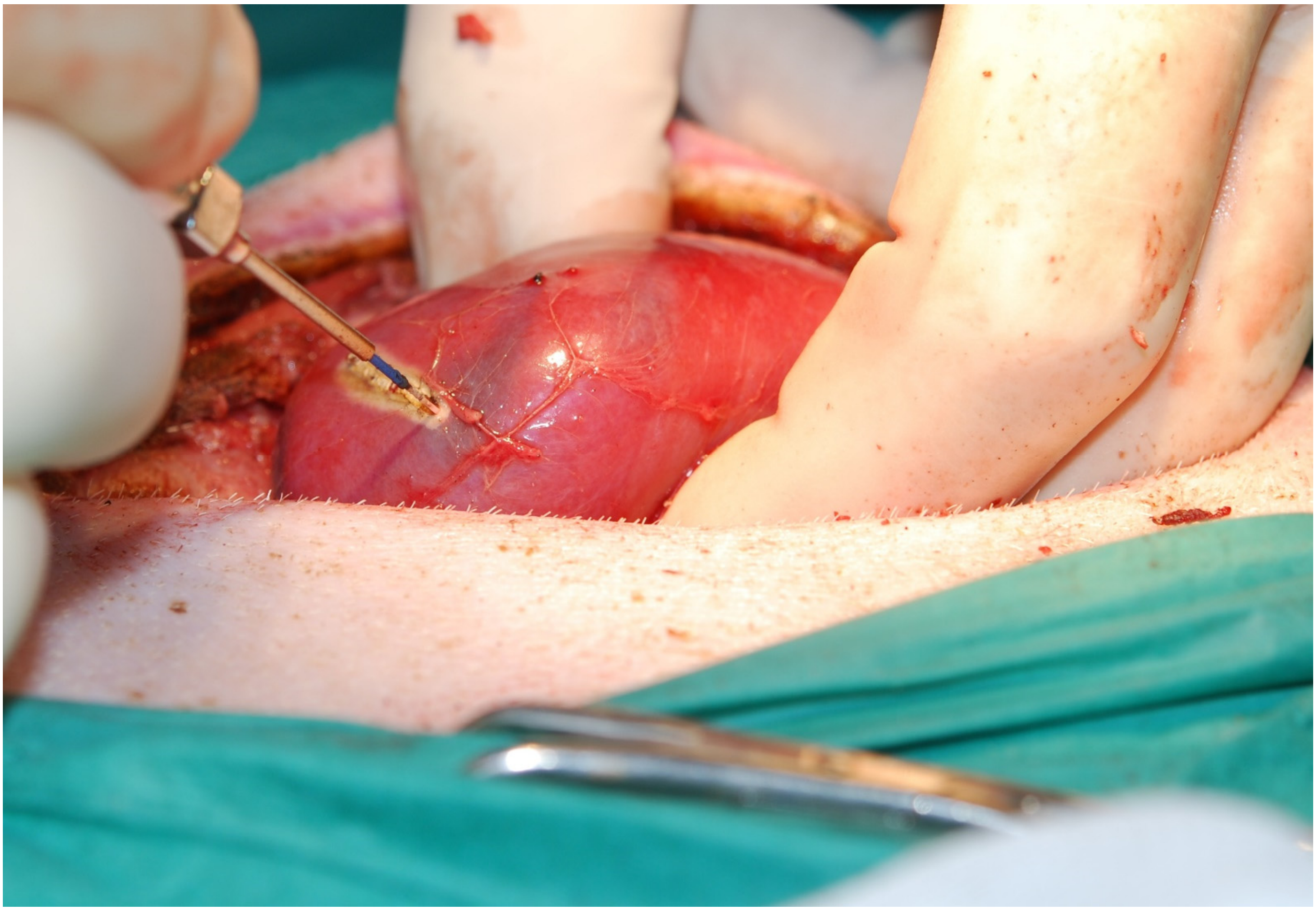

Figure S1. Kidney incision with a thulium-doped fiber laser.

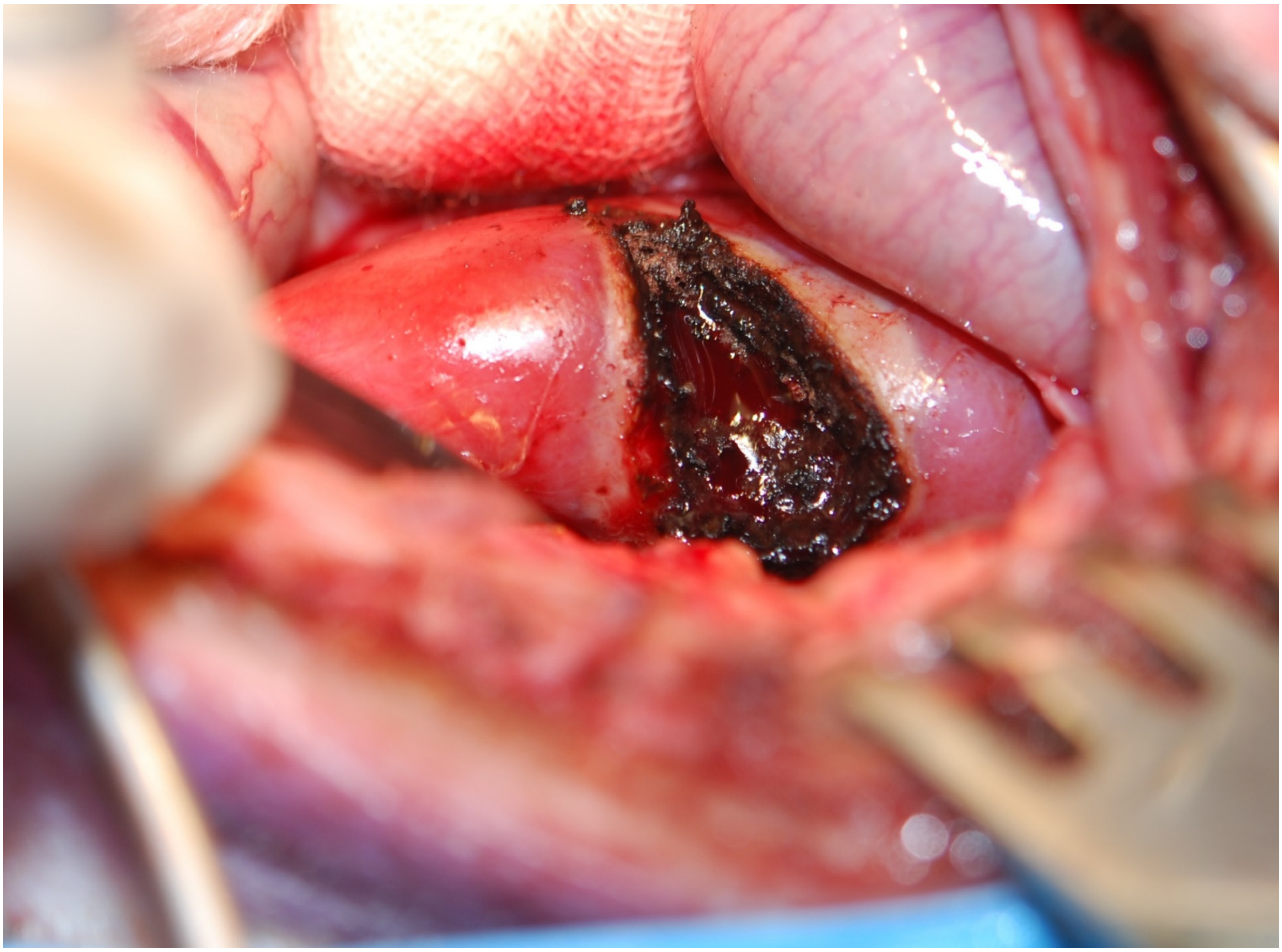

Figure S2. Porcine kidney after laser partial nephrectomy.
